# Change-point analysis data of neonatal diffusion tensor MRI in preterm and term-born infants

**DOI:** 10.1016/j.dib.2017.04.020

**Published:** 2017-04-20

**Authors:** Dan Wu, Linda Chang, Kentaro Akazawa, Kumiko Oishi, Jon Skranes, Thomas Ernst, Kenichi Oishi

**Affiliations:** a Department of Radiology, Johns Hopkins University School of Medicine, Baltimore, MD, USA; b Department of Medicine, School of Medicine, University of Hawaii at Manoa, Honolulu, HI, USA; c Department of Biomedical Engineering, Johns Hopkins University, Baltimore, MD, USA; d Department of Laboratory Medicine, Children׳s and Women׳s Health, Norwegian University of Science and Technology, Trondheim, Norway

**Keywords:** Neonatal brain MRI, Preterm-born infants, Change-point analysis, Radial diffusivity, Axial diffusivity

## Abstract

The data presented in this article are related to the research article entitled “Mapping the Critical Gestational Age at Birth that Alters Brain Development in Preterm-born Infants using Multi-Modal MRI” (Wu et al., 2017) [1]. Brain immaturity at birth poses critical neurological risks in the preterm-born infants. We used a novel change-point model to analyze the critical gestational age at birth (GAB) that could affect postnatal development, based on diffusion tensor MRI (DTI) acquired from 43 preterm and 43 term-born infants in 126 brain regions. In the corresponding research article, we presented change-point analysis of fractional anisotropy (FA) and mean diffusivities (MD) measurements in these infants. In this article, we offered the relative changes of axonal and radial diffusivities (AD and RD) in relation to the change of FA and FA-based change-points, and we also provided the AD- and RD-based change-point results.

**Specifications Table**

**Table:** 

Subject area	*NeuroImaging*
More specific subject area	*Neonatal brain MRI, Image analysis*
Type of data	*Figures*
How data was acquired	*Diffusion tensor MRI was acquired using a 3.0 T Siemens TIM Trio scanner*
Data format	*Analyzed*
Experimental factors	*Data from 43 term-born and 43 preterm-born infants were used in the analysis*
Experimental features	*The MRI data were first segmented to 126 brain regions with automated atlas-based image segmentation, and then the metadata from each region were fitted by a multivariate linear change-point model.*
Data source location	*Queen׳s Medical Center, University of Hawaii, Honolulu, Hawaii, USA*
Data accessibility	*Data is within this article.*

**Value of the data**

• The relative changes of AD and RD with GAB lead to distinct change patterns in FA before and after the change-points.
• The change-point analysis of AD and RD data offers complementary information to the change-points in FA and MD.
• The change-point model characterizes features of the brain developmental trajectory, and the whole map of change-points in 126 structures demonstrate regional variations of brain development associated with preterm-term birth.

## 1 Data

### 1.1 GAB-dependent changes of RD and AD in relation with FA

**Fig. 1:**
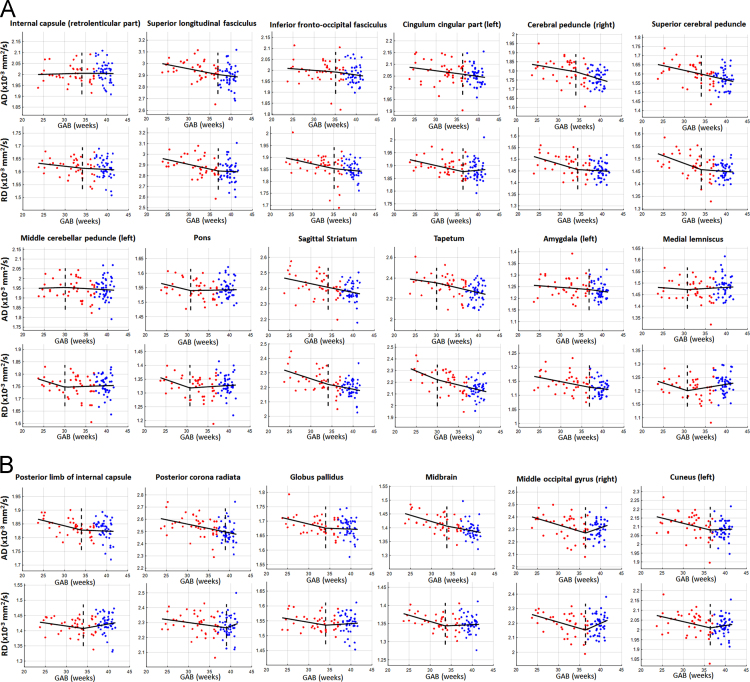
The change of axial diffusivity (AD) and radial diffusivity (RD) with GAB in relation to the GAB-dependent changes in FA (Fig. 1 in reference [1]). The red and blue dots denote data from preterm and term-born neonates, respectively, after correcting for PMA at scan and gender. The change points that were derived from FA data (dashed vertical lines) are applied to multivariate linear regression analysis of AD and RD, according to the change point model in [1]. The black lines show the fitting results of AD and RD, using the FA-based change points in the change-point model, with only the GAB-dependent terms (without other covariates). The structures shown in A and B followed two different changing patterns, which correspond to Fig. 1A and B in reference [1].

The change of FA is driven by the relative change of axial and radial diffusivities (AD and RD). Two different patterns of GAB-dependent FA changes were demonstrated in Fig. 1 of the related paper [1]. Therefore, we plotted the changes of AD and RD with respect to GAB, in the structures corresponding to Fig. 1 in the research paper [1]. The red and blue dots in Fig. 1 denote data from preterm and term-born neonates, respectively, after correcting for PMA at scan and gender. The dashed vertical lines indicate the change points analyzed using the FA data. The structures shown in Fig. 1A (correspond to the first pattern in Fig. 1A in [1]) exhibited the following characteristics: 1a) increased FA with GAB before the change-points and small changes thereafter; and 1b) faster rate of decrease in RD with GAB compared to that in AD before the change-points, and similar rates of decrease in RD and AD thereafter. Conversely, the structures shown in Fig. 1B (correspond to the second patter in Figure 1B in [1]) demonstrated: 2a) relatively stable FA before the change-points which decreased with GAB thereafter; and 2b) similar rates of decrease in AD and RD before the change-points, and slow decrease of AD and slight increase of RD after the change-points.

### 1.2 AD- and RD-based change-point analyses

**Fig. 2:**
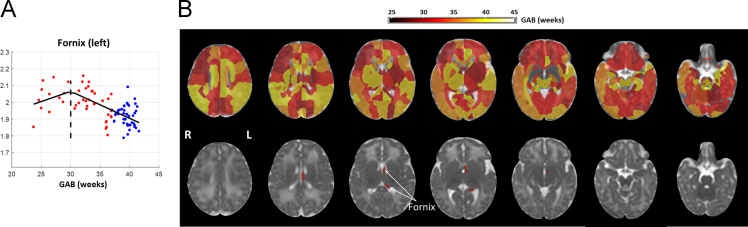
Change-point analysis of the AD data. (A) The left fornix showed significant change-point (familywise p=0.01) at GAB of 30 weeks. The x-axis represents GAB in unit of weeks, and the y-axis represents AD values after correcting for PMA at scan and gender, based on the change point model. The red and blue dots denote data from preterm and term-born neonates, respectively. The black line shows fitted AD values based on the GAB-dependent terms in the change point model in reference [1], and dashed line indicates the position of change point. (B) Whole brain maps of AD-based change-points overlaid on the JHU-neonate MD atlas, with (top row) and without (bottom row) the significance threshold (familywise p<0.05).

**Fig. 3:**
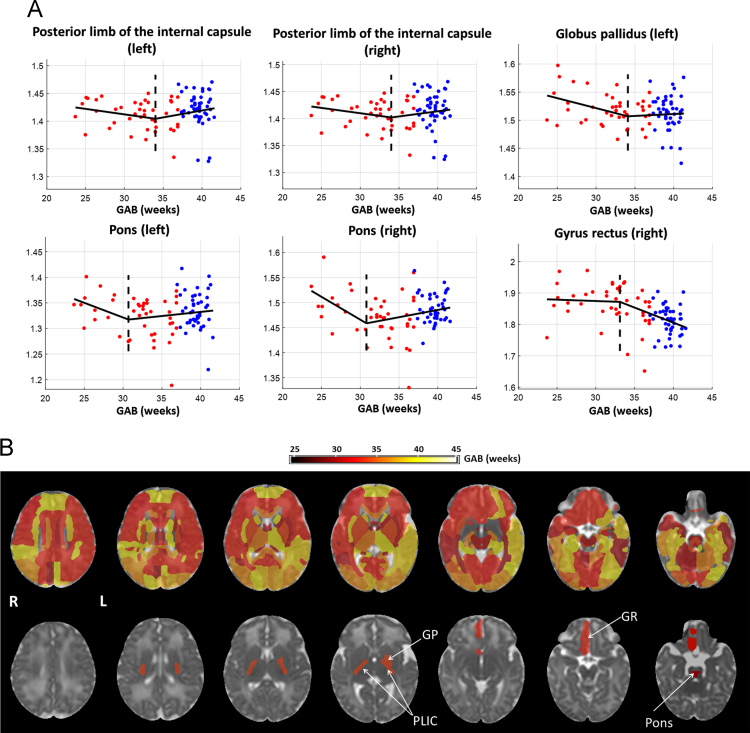
Change-point analysis of the RD data. (A) Six brain regions showed significant change-point (familywise p<0.05) with GAB between 30–34 weeks. The red and blue dots denote data from preterm and term-born neonates, respectively, after correcting for PMA at scan and gender. The black line shows fitted RD values based on the GAB-dependent terms in the change point model in reference [1], and dashed line indicates the position of change point. (B) Whole brain maps of RD-based change-points overlaid on the JHU-neonate MD atlas, with (top row) and without (bottom row) the significance threshold (familywise p<0.05). Abbreviations: GP –globus pallidus; PLIC –posterior limb of internal capsule; RG –gyrus rectus.

Change-point analysis was performed on the AD and RD data in 126 brain structures, and the change-points detected in individual regions were mapped onto an MD image from the JHU-neonatal atlas in Figs. 2B and 3B, respectively. The AD-based change-point analysis only showed significance (familywise p = 0.01) in the left fornix at GAB of 30 weeks (Fig. 2A, and bottom row in Fig. 2B), where the AD values increased with GAB before the change-point and then decreased after the change-point. The RD-based change point analysis showed significance (familywise p<0.05) in six brain regions (Fig. 3A, and bottom row in Fig. 3B), including the bilateral posterior limb of the internal capsule (PLIC), the left globus pallidus (GP), the bilateral pons, and the right gyrus rectus (RG). The first five structures showed similar patterns, where RD decreased with GAB before the change-points, and remained relatively stable thereafter; whereas right RG demonstrated an opposite pattern, where RD was stable before the change-point and then decreased with GAB thereafter. Among these six regions, the pons showed earliest change-points around GAB of 31 weeks, followed by the right RG at 33 weeks, and the other structures around 34 weeks.

## 2 Experimental design, materials and methods

### 2.1 Participants

The Infants were recruited and scanned at the University of Hawaii and the Queen׳s Medical Center MR Research Center in Honolulu, HI. The infants׳ parents or legal guardians provided written and verbal informed consent for the study, which was approved by the Cooperative Institutional Review Board of the Queen׳s Medical Center and the University of Hawaii, and the Johns Hopkins University. Participants were screened by telephone initially and again by a physician on the day of the scans for the following criteria. They were excluded if the mothers were <18 years of age, or were unable to fully understand English, which would have precluded informed consent. Inclusion criteria for the infants were: 1) new born male or female child of any ethnicity; 2) born prematurely at <37 weeks gestational weeks (for the preterm infants) or born at 37–42 weeks gestation (for term born infants); 3) had parental/legal guardian consents. Exclusion criteria for the term-born infants included: 1) prolonged intensive care (>7 days); 2) intracranial hemorrhage; 3) neonatal hypoxic-ischemic encephalopathy; 4) known toxoplasmosis; other (syphilis, varicella-zoster, parvovirus B19); and rubella, Cytomegalovirus (CMV), and Herpes infections (TORCH); and 5) congenital heart disease or other anomaly; or 6) any chromosomal anomaly. Preterm-born infants were excluded if they 1) required supplementary oxygen or mechanical ventilation during the time of scanning; 2) had a circulatory, respiratory or airway abnormality; or 3) were diagnosed with fever, epilepsy, or active infection. All infants were also excluded if they had any contraindications for MR studies (e.g., metallic or electronic implants).

After all exclusion criteria, 43 preterm-born infants (24 male and 19 female), with GABs ranging from 23.7–36.9 weeks, and 43 term-born infants (21 male and 22 female), with GABs ranging from 38.0–41.6 weeks, were used in the analysis. The Infants were scanned at an estimated postmenstrual age (PMA) around 43 weeks. Other demographic and clinical information of the infants can be found from Table 1 in reference [1].

### 2.2 MRI data acquisition

The infants were scanned without sedation, on a 3.0 T Siemens TIM Trio scanner (Siemens Medical Solutions, Erlangen, Germany). The DTI images were acquired with single-shot EPI sequence at: field-of-view (FOV) = 160 × 160 mm, in-plane resolution = 2×2 mm, 40–50 axial with slice thickness of 2.5 mm, echo time (TE) = 106 ms and repetition time (TR) = 7–9 s, 12 diffusion directions with *b* = 1000 s/mm^2^ and one minimally diffusion-weighted (b0) image. T2 mapping data was acquired with a dual-echo fast spin-echo sequence at: FOV = 250 × 250 mm, in-plane resolution=1.95×1.95 mm, 40–50 axial slices with slice thickness of 2.5 mm thickness, and TR/TE1/TE2 = 4550/24/130 ms.

### 2.3 MRI data analysis

#### 2.3.1 Atlas-based image segmentation

The FA and MD images were reconstructed from the DTI data, and then transformed to the JHU-neonate single brain DTI atlas [2], using a linear transformation followed by Large Deformation Diffeomorphic Metric Mapping (LDDMM) [3], [4]. Through this procedure, the brains were segmented to 126 regions of interests (ROIs), as defined in the JHU neonatal DTI atlas [2]. T2 relaxation-time maps were calculated by log-linear fitting of the dual-echo T2 mapping data. The T2 mapping data were first skull-stripped, and then the skull-stripped second-echo T2-weighted image was transformed to b0 image of the DTI data, using linear and LDDMM transformations. Through these cascading steps, the T2 maps can be transformed to the JHU-neonate atlas and segmented to the same 126 ROIs.

#### 2.3.2 Change point analysis

The change-point model describes the change of MRI measurements by two linear phases with respect to GAB, with the transition point being the “change point”. The model regresses MRI measurements (*y*) on the multiple covariates, including the absolute change of GAB (*g*), the change-point (*Δ*) dependent change of GAB, as well as the PMA at scan (*p*) and sex (*s*), as follows

$$y_{k}=a_{0}+a_{1}\cdot g_{k}+a_{2}\cdot (g_{k}-∆)\cdot H(g_{k}-∆)+a_{3}\cdot p_{k}+a_{4}\cdot s_{k}+\epsilon_{k}$$

where *H* is an indicator function with *H*(*g*_*k*_−*Δ*)=1 if *g*_*k*_>*Δ* and *H*(*g*_*k*_−*Δ*)=0 otherwise. The mathematical procedures of model fitting can be found in reference [1]. Once an optimal change-point was found, we performed to permutation test [5] to evaluate the significance of the change-point, and bootstrap [6], [7] to evaluated the standard deviation of estimated change-point [1].
