# Supplementary material for: Change-point analysis data of neonatal diffusion tensor MRI in preterm and term-born infants
Source: Data Brief. 2017 Apr 20;12:453–8. doi: 10.1016/j.dib.2017.04.020 (PMC5426014; doi:10.1016/j.dib.2017.04.020)
Supplement: Supplementary file 1 — Supplementary material [file mmc1.docx]

None.
